# Crystal structure of benzimidazolium salicylate

**DOI:** 10.1107/S2056989015017764

**Published:** 2015-09-26

**Authors:** M. Amudha, P. Praveen Kumar, G. Chakkaravarthi

**Affiliations:** a Department of physics, Presidency College, Chennai 600 005, India; b Department of Physics, Aalim Muhammed Salegh College of Engineering, Chennai 600 055, India; c Department of Physics, CPCL Polytechnic College, Chennai 600 068, India

**Keywords:** crystal structure, benzimidazolium, salicylate, hydrogen bonding

## Abstract

In the anion of the title mol­ecular salt, C_7_H_7_N_2_^+^·C_7_H_5_O_3_^−^ (systematic name: 1*H*-benzimidazol-3-ium 2-hy­droxy­ben­zo­ate), there is an intra­molecular O—H⋯O hydrogen bond that generates an *S*(6) ring motif. The CO_2_ group makes a dihedral angle of 5.33 (15)° with its attached ring. In the crystal, the dihedral angle between the benzimidazolium ring and the anion benzene ring is 75.88 (5)°. Two cations bridge two anions *via* two pairs of N—H⋯O hydrogen bonds, enclosing an *R* ^4^_4_(16) ring motif, forming a four-membered centrosymmetric arrangement. These units are linked *via* C—H⋯O hydrogen bonds, forming chains propagating along the *b-*axis direction. The chains are linked by C—H⋯π and π–π inter­actions [inter-centroid distances = 3.4156 (7) and 3.8196 (8) Å], forming a three-dimensional structure.

## Related literature   

For biological applications of benzimidazole derivatives, see: Narasimhan *et al.* (2012 ▸). For related structures, see: Ennajih *et al.* (2010 ▸); Haque *et al.* (2012 ▸); Mani *et al.* (2015 ▸).
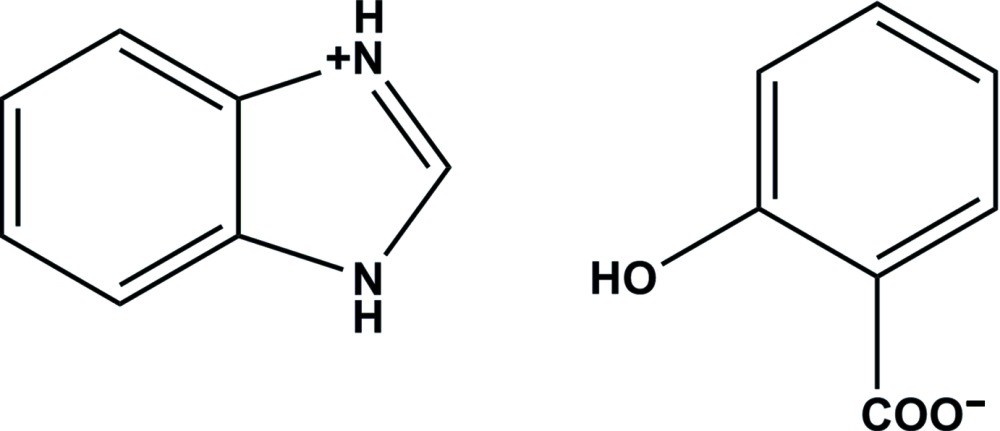


## Experimental   

### Crystal data  

C_7_H_7_N_2_^+^·C_7_H_5_O_3_^−^
*M* *_r_* = 256.26
Monoclinic, 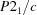

*a* = 7.4776 (3) Å
*b* = 6.7002 (2) Å
*c* = 24.9017 (9) Å
β = 94.445 (2)°
*V* = 1243.86 (8) Å^3^
*Z* = 4
Mo *K*α radiation
μ = 0.10 mm^−1^
*T* = 295 K
0.34 × 0.30 × 0.25 mm

### Data collection  

Bruker Kappa APEXII CCD diffractometer
Absorption correction: multi-scan (*SADABS*; Sheldrick, 1996 ▸) *T*_min_ = 0.967, *T*_max_ = 0.976
23125 measured reflections
4606 independent reflections
3020 reflections with *I* > 2σ(*I*)
*R*_int_ = 0.024

### Refinement  

*R*[*F* ^2^ > 2σ(*F* ^2^)] = 0.048
*wR*(*F* ^2^) = 0.142
*S* = 1.03
4606 reflections
176 parameters
1 restraint
H atoms treated by a mixture of independent and constrained refinement
Δρ_max_ = 0.35 e Å^−3^
Δρ_min_ = −0.26 e Å^−3^

### 

Data collection: *APEX2* (Bruker, 2004 ▸); cell refinement: *SAINT* (Bruker, 2004 ▸); data reduction: *SAINT*; program(s) used to solve structure: *SHELXS97* (Sheldrick, 2008 ▸); program(s) used to refine structure: *SHELXL97* (Sheldrick, 2008 ▸); molecular graphics: *PLATON* (Spek, 2009 ▸); software used to prepare material for publication: *SHELXL97* and *PLATON*.

## Supplementary Material

Crystal structure: contains datablock(s) global, I. DOI: 10.1107/S2056989015017764/su5212sup1.cif

Structure factors: contains datablock(s) I. DOI: 10.1107/S2056989015017764/su5212Isup2.hkl

Supporting information file. DOI: 10.1107/S2056989015017764/su5212Isup3.cml

**Fig. 1.**
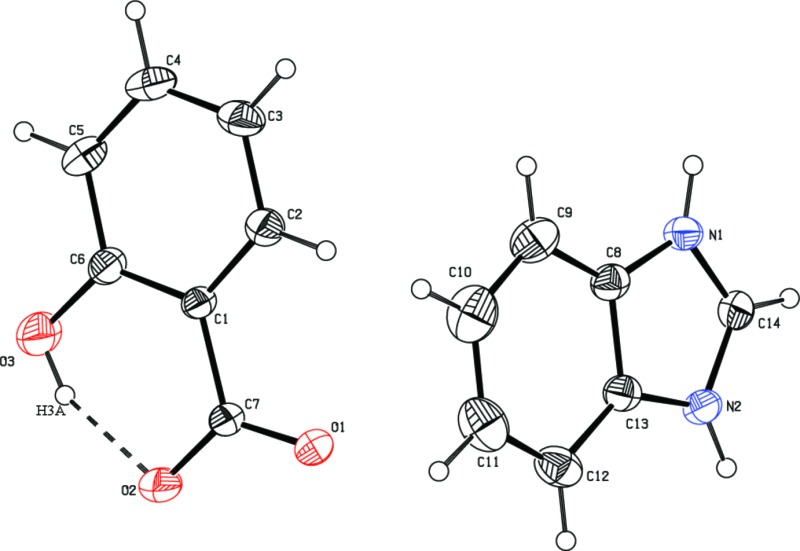
The molecular structure of the title salt, with atom labelling. The displacement ellipsoids are drawn at the 30% probability level.

**Fig. 2.**
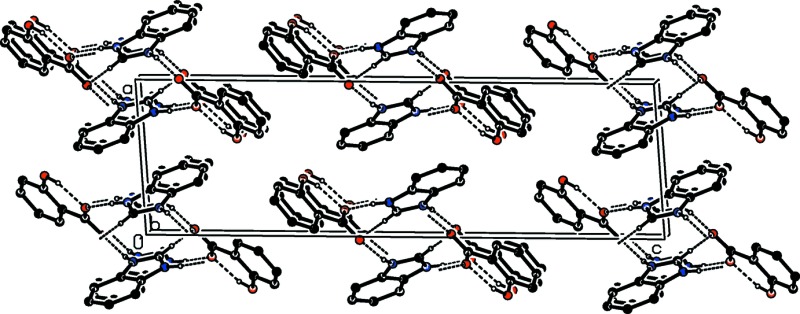
The crystal packing of the title molecular salt, viewed along the b axis. The N—H···O and C—H···O hydrogen bonds are shown as dashed lines (see Table 1). H atoms not involved in these interactions have been omitted for clarity.

. DOI: 10.1107/S2056989015017764/su5212fig1.tif
The mol­ecular structure of the title salt, with atom labelling. The displacement ellipsoids are drawn at the 30% probability level.

b . DOI: 10.1107/S2056989015017764/su5212fig2.tif
The crystal packing of the title mol­ecular salt, viewed along the *b* axis. The N—H⋯O and C—H⋯O hydrogen bonds are shown as dashed lines (see Table 1). H atoms not involved in these inter­actions have been omitted for clarity.

CCDC reference: 1426331

Additional supporting information:  crystallographic information; 3D view; checkCIF report

## Figures and Tables

**Table 1:** Hydrogen-bond geometry (, ) *Cg*3 is the centroid of the C1C6 ring.

*D*H*A*	*D*H	H*A*	*D* *A*	*D*H*A*
O3H3*A*O2	0.83(1)	1.78(1)	2.5425(14)	152(2)
N1H1*A*O1^i^	0.86	1.81	2.6139(13)	155
N2H2*A*O2^ii^	0.86	1.81	2.6448(13)	164
C14H14O1^iii^	0.93	2.22	3.1161(16)	161
C3H3*Cg*3^iv^	0.93	2.81	3.5779(15)	141
C10H10*Cg*3^v^	0.93	2.88	3.6302(17)	139

Symmetry codes: (i) 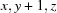; (ii) 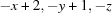; (iii) 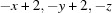; (iv) 
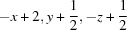
; (v) 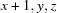.

## supplementary crystallographic information

### S1. Structural commentary

Benzimidazoles and their derivatives have diverse biological and clinical applications (Narasimhan *et al.*, 2012).

The molecular structure of the title salt is illustrated in Fig. 1. The geometric parameters are comparable with those reported for similar structures (Ennajih *et al.*, 2010; Haque *et al.*, 2012; Mani *et al.*, 2015). The molecular structure of the anion is stabilized by an intra­molecular O—H···O hydrogen bond which generates an S(6) ring motif (Table 1 and Fig. 1).

In the crystal, the dihedral angle between the nine-membered benzimidazolium ring (C8—C13/N2/C14/N1) and the anion benzene ring (C1—C6) is 75.88 (5)°. Two cations bridge two anions via two pairs of N—H···O hydrogen bonds, enclosing an R^4^_4_(16) ring motif, forming a four-membered centrosymmetric arrangement (Table 1 and Fig. 2). These units are linked via C—H···O hydrogen bonds forming chains along the *b* axis direction. The chains are linked by C—H···π (Table 1) and π···π inter­actions [Cg1···Cg1^i^ = 3.4156 (7) Å; Cg1···Cg2^ii^ = 3.8196 (8) Å; Cg1 and Cg2 are the centroids of rings (N1/C8/C13/N2/C14) and (C8—C13), respectively; symmetry codes: (i) x+2, y+2, -z; (ii) -x+3, -y+2, -z], forming a three-dimensional structure.

### S2. Synthesis and crystallization

Benzimidazole (6 g) and salicylic acid (7.002 g) were dissolved in an equimolar ratio in methanol and stirred well for *ca* 6 h. The saturated solution was filtered and allowed to evaporate slowly at room temperature. Colourless block-shaped crystals of the title compound were obtained within seven days.

### S3. Refinement

Crystal data, data collection and structure refinement details are summarized in Table 2. The hydroxyl H atom was located in a difference Fourier map and refined with a distance restraint: O—H = 0.82 (1) Å with U_iso_(H) = 1.5U_eq_(O). The NH and C-bound H atoms were positioned geometrically and refined using a riding model: N—H = 0.86 Å, C—H = 0.93 Å with U_iso_(H) = 1.2U_eq_(N,C).

### Crystal data

**Table:**

C_7_H_7_N_2_^+^·C_7_H_5_O_3_^−^	*F*(000) = 536
*M**_r_* = 256.26	*D*_x_ = 1.368 Mg m^−^^3^
Monoclinic, *P*2_1_/*c*	Mo *K*α radiation, λ = 0.71073 Å
Hall symbol: -P 2ybc	Cell parameters from 8207 reflections
*a* = 7.4776 (3) Å	θ = 2.7–31.0°
*b* = 6.7002 (2) Å	µ = 0.10 mm^−^^1^
*c* = 24.9017 (9) Å	*T* = 295 K
β = 94.445 (2)°	Block, colourless
*V* = 1243.86 (8) Å^3^	0.34 × 0.30 × 0.25 mm
*Z* = 4	

### Data collection

**Table:**

Bruker Kappa APEXII CCD diffractometer	4606 independent reflections
Radiation source: fine-focus sealed tube	3020 reflections with *I* > 2σ(*I*)
Graphite monochromator	*R*_int_ = 0.024
ω and φ scan	θ_max_ = 39.4°, θ_min_ = 2.7°
Absorption correction: multi-scan (*SADABS*; Sheldrick, 1996)	*h* = −10→10
*T*_min_ = 0.967, *T*_max_ = 0.976	*k* = −11→9
23125 measured reflections	*l* = −35→35

### Refinement

**Table:**

Refinement on *F*^2^	Secondary atom site location: difference Fourier map
Least-squares matrix: full	Hydrogen site location: inferred from neighbouring sites
*R*[*F*^2^ > 2σ(*F*^2^)] = 0.048	H atoms treated by a mixture of independent and constrained refinement
*wR*(*F*^2^) = 0.142	*w* = 1/[σ^2^(*F*_o_^2^) + (0.0618*P*)^2^ + 0.2364*P*] where *P* = (*F*_o_^2^ + 2*F*_c_^2^)/3
*S* = 1.03	(Δ/σ)_max_ < 0.001
4606 reflections	Δρ_max_ = 0.35 e Å^−^^3^
176 parameters	Δρ_min_ = −0.26 e Å^−^^3^
1 restraint	Extinction correction: *SHELXL*, Fc^*^=kFc[1+0.001xFc^2^λ^3^/sin(2θ)]^-1/4^
Primary atom site location: structure-invariant direct methods	Extinction coefficient: 0.025 (3)

### Special details

**Table:**

Geometry. All esds (except the esd in the dihedral angle between two l.s. planes) are estimated using the full covariance matrix. The cell esds are taken into account individually in the estimation of esds in distances, angles and torsion angles; correlations between esds in cell parameters are only used when they are defined by crystal symmetry. An approximate (isotropic) treatment of cell esds is used for estimating esds involving l.s. planes.
Refinement. Refinement of F^2^ against ALL reflections. The weighted R-factor wR and goodness of fit S are based on F^2^, conventional R-factors R are based on F, with F set to zero for negative F^2^. The threshold expression of F^2^ > 2sigma(F^2^) is used only for calculating R-factors(gt) etc. and is not relevant to the choice of reflections for refinement. R-factors based on F^2^ are statistically about twice as large as those based on F, and R- factors based on ALL data will be even larger.

### Fractional atomic coordinates and isotropic or equivalent isotropic displacement parameters (Å^2^)

**Table:**

	*x*	*y*	*z*	*U*_iso_*/*U*_eq_	
C1	0.85922 (14)	0.54826 (15)	0.16407 (4)	0.0321 (2)	
C2	0.94451 (17)	0.73153 (18)	0.17277 (5)	0.0414 (3)	
H2	1.0347	0.7692	0.1511	0.050*	
C3	0.8971 (2)	0.8581 (2)	0.21304 (5)	0.0499 (3)	
H3	0.9554	0.9798	0.2187	0.060*	
C4	0.76260 (19)	0.8026 (2)	0.24480 (5)	0.0503 (3)	
H4	0.7296	0.8882	0.2717	0.060*	
C5	0.67701 (18)	0.6234 (2)	0.23729 (5)	0.0475 (3)	
H5	0.5866	0.5878	0.2591	0.057*	
C6	0.72508 (16)	0.49396 (18)	0.19710 (4)	0.0391 (3)	
C7	0.91104 (17)	0.41505 (16)	0.12000 (4)	0.0375 (2)	
C8	1.27512 (15)	1.05724 (17)	0.05515 (5)	0.0381 (2)	
C9	1.3448 (2)	1.0252 (2)	0.10764 (6)	0.0562 (4)	
H9	1.3355	1.1203	0.1345	0.067*	
C10	1.4287 (2)	0.8448 (3)	0.11783 (7)	0.0697 (5)	
H10	1.4781	0.8180	0.1525	0.084*	
C11	1.4420 (2)	0.7016 (3)	0.07804 (8)	0.0659 (4)	
H11	1.4981	0.5810	0.0870	0.079*	
C12	1.37525 (18)	0.7325 (2)	0.02618 (6)	0.0516 (3)	
H12	1.3854	0.6367	−0.0004	0.062*	
C13	1.29132 (15)	0.91474 (16)	0.01517 (5)	0.0371 (2)	
C14	1.14314 (16)	1.17020 (18)	−0.02020 (5)	0.0409 (3)	
H14	1.0797	1.2524	−0.0450	0.049*	
N1	1.18155 (14)	1.21523 (14)	0.03109 (4)	0.0398 (2)	
H1A	1.1532	1.3239	0.0467	0.048*	
N2	1.20728 (13)	0.99240 (14)	−0.03137 (4)	0.0400 (2)	
H2A	1.1981	0.9351	−0.0624	0.048*	
O1	1.02203 (14)	0.47695 (13)	0.08885 (4)	0.0521 (3)	
O2	0.83774 (17)	0.24546 (14)	0.11610 (4)	0.0613 (3)	
O3	0.63621 (17)	0.31901 (17)	0.19135 (4)	0.0653 (3)	
H3A	0.680 (3)	0.261 (3)	0.1658 (7)	0.098*	

### Atomic displacement parameters (Å^2^)

**Table:**

	*U*^11^	*U*^22^	*U*^33^	*U*^12^	*U*^13^	*U*^23^
C1	0.0371 (5)	0.0327 (5)	0.0266 (4)	0.0025 (4)	0.0035 (4)	−0.0024 (4)
C2	0.0442 (6)	0.0390 (6)	0.0411 (6)	−0.0041 (5)	0.0050 (5)	−0.0034 (4)
C3	0.0594 (8)	0.0395 (6)	0.0497 (7)	−0.0019 (6)	−0.0034 (6)	−0.0137 (5)
C4	0.0556 (7)	0.0559 (8)	0.0389 (6)	0.0139 (6)	0.0004 (5)	−0.0169 (5)
C5	0.0454 (6)	0.0626 (8)	0.0357 (6)	0.0047 (6)	0.0106 (5)	−0.0078 (5)
C6	0.0413 (6)	0.0431 (6)	0.0334 (5)	−0.0024 (5)	0.0065 (4)	−0.0031 (4)
C7	0.0508 (6)	0.0334 (5)	0.0290 (5)	0.0041 (4)	0.0074 (4)	−0.0012 (4)
C8	0.0342 (5)	0.0409 (6)	0.0400 (6)	−0.0039 (4)	0.0077 (4)	−0.0050 (4)
C9	0.0551 (8)	0.0698 (9)	0.0426 (7)	−0.0019 (7)	−0.0021 (6)	−0.0076 (6)
C10	0.0627 (9)	0.0871 (12)	0.0566 (9)	0.0073 (9)	−0.0127 (7)	0.0111 (8)
C11	0.0541 (8)	0.0583 (9)	0.0838 (11)	0.0114 (7)	−0.0049 (8)	0.0125 (8)
C12	0.0431 (7)	0.0421 (6)	0.0701 (9)	0.0045 (5)	0.0083 (6)	−0.0051 (6)
C13	0.0321 (5)	0.0367 (5)	0.0433 (6)	−0.0031 (4)	0.0089 (4)	−0.0040 (4)
C14	0.0436 (6)	0.0393 (6)	0.0409 (6)	−0.0013 (5)	0.0113 (5)	0.0021 (4)
N1	0.0439 (5)	0.0342 (5)	0.0426 (5)	−0.0009 (4)	0.0119 (4)	−0.0064 (4)
N2	0.0454 (5)	0.0404 (5)	0.0354 (5)	−0.0036 (4)	0.0100 (4)	−0.0066 (4)
O1	0.0708 (6)	0.0415 (5)	0.0480 (5)	0.0047 (4)	0.0301 (4)	−0.0018 (4)
O2	0.0956 (8)	0.0428 (5)	0.0487 (5)	−0.0176 (5)	0.0264 (5)	−0.0168 (4)
O3	0.0751 (7)	0.0598 (6)	0.0656 (7)	−0.0275 (5)	0.0340 (5)	−0.0142 (5)

### Geometric parameters (Å, º)

**Table:**

C1—C2	1.3929 (16)	C8—C13	1.3913 (16)
C1—C6	1.3939 (15)	C9—C10	1.376 (2)
C1—C7	1.4889 (14)	C9—H9	0.9300
C2—C3	1.3803 (17)	C10—C11	1.388 (3)
C2—H2	0.9300	C10—H10	0.9300
C3—C4	1.378 (2)	C11—C12	1.364 (2)
C3—H3	0.9300	C11—H11	0.9300
C4—C5	1.367 (2)	C12—C13	1.3902 (17)
C4—H4	0.9300	C12—H12	0.9300
C5—C6	1.3922 (16)	C13—N2	1.3769 (15)
C5—H5	0.9300	C14—N2	1.3219 (15)
C6—O3	1.3496 (15)	C14—N1	1.3220 (15)
C7—O1	1.2499 (14)	C14—H14	0.9300
C7—O2	1.2620 (14)	N1—H1A	0.8600
C8—N1	1.3798 (15)	N2—H2A	0.8600
C8—C9	1.3861 (18)	O3—H3A	0.834 (9)
			
C2—C1—C6	118.68 (10)	C10—C9—H9	121.9
C2—C1—C7	120.07 (10)	C8—C9—H9	121.9
C6—C1—C7	121.25 (10)	C9—C10—C11	122.17 (14)
C3—C2—C1	120.96 (12)	C9—C10—H10	118.9
C3—C2—H2	119.5	C11—C10—H10	118.9
C1—C2—H2	119.5	C12—C11—C10	121.97 (14)
C4—C3—C2	119.41 (12)	C12—C11—H11	119.0
C4—C3—H3	120.3	C10—C11—H11	119.0
C2—C3—H3	120.3	C11—C12—C13	116.52 (13)
C5—C4—C3	120.90 (11)	C11—C12—H12	121.7
C5—C4—H4	119.5	C13—C12—H12	121.7
C3—C4—H4	119.5	N2—C13—C12	131.86 (11)
C4—C5—C6	120.08 (12)	N2—C13—C8	106.47 (10)
C4—C5—H5	120.0	C12—C13—C8	121.64 (12)
C6—C5—H5	120.0	N2—C14—N1	110.72 (11)
O3—C6—C5	117.70 (11)	N2—C14—H14	124.6
O3—C6—C1	122.34 (10)	N1—C14—H14	124.6
C5—C6—C1	119.96 (11)	C14—N1—C8	108.01 (10)
O1—C7—O2	123.80 (10)	C14—N1—H1A	126.0
O1—C7—C1	118.80 (10)	C8—N1—H1A	126.0
O2—C7—C1	117.40 (10)	C14—N2—C13	108.22 (10)
N1—C8—C9	132.02 (11)	C14—N2—H2A	125.9
N1—C8—C13	106.57 (10)	C13—N2—H2A	125.9
C9—C8—C13	121.41 (12)	C6—O3—H3A	105.2 (16)
C10—C9—C8	116.28 (14)		
			
C6—C1—C2—C3	−0.37 (17)	C13—C8—C9—C10	−0.6 (2)
C7—C1—C2—C3	179.30 (11)	C8—C9—C10—C11	−0.5 (2)
C1—C2—C3—C4	−0.42 (19)	C9—C10—C11—C12	1.1 (3)
C2—C3—C4—C5	0.6 (2)	C10—C11—C12—C13	−0.6 (2)
C3—C4—C5—C6	−0.1 (2)	C11—C12—C13—N2	−178.38 (13)
C4—C5—C6—O3	−179.93 (12)	C11—C12—C13—C8	−0.51 (19)
C4—C5—C6—C1	−0.75 (19)	N1—C8—C13—N2	0.10 (12)
C2—C1—C6—O3	−179.91 (12)	C9—C8—C13—N2	179.49 (11)
C7—C1—C6—O3	0.42 (18)	N1—C8—C13—C12	−178.25 (11)
C2—C1—C6—C5	0.96 (17)	C9—C8—C13—C12	1.14 (18)
C7—C1—C6—C5	−178.71 (11)	N2—C14—N1—C8	−0.50 (13)
C2—C1—C7—O1	−5.18 (17)	C9—C8—N1—C14	−179.06 (14)
C6—C1—C7—O1	174.49 (11)	C13—C8—N1—C14	0.23 (13)
C2—C1—C7—O2	175.54 (12)	N1—C14—N2—C13	0.56 (13)
C6—C1—C7—O2	−4.79 (17)	C12—C13—N2—C14	177.72 (13)
N1—C8—C9—C10	178.60 (14)	C8—C13—N2—C14	−0.40 (12)

### Hydrogen-bond geometry (Å, º)

Cg3 is the centroid of the C1–C6 ring.

**Table:**

*D*—H···*A*	*D*—H	H···*A*	*D*···*A*	*D*—H···*A*
O3—H3*A*···O2	0.83 (1)	1.78 (1)	2.5425 (14)	152 (2)
N1—H1*A*···O1^i^	0.86	1.81	2.6139 (13)	155
N2—H2*A*···O2^ii^	0.86	1.81	2.6448 (13)	164
C14—H14···O1^iii^	0.93	2.22	3.1161 (16)	161
C3—H3···*Cg*3^iv^	0.93	2.81	3.5779 (15)	141
C10—H10···*Cg*3^v^	0.93	2.88	3.6302 (17)	139

Symmetry codes: (i) *x*, *y*+1, *z*; (ii) −*x*+2, −*y*+1, −*z*; (iii) −*x*+2, −*y*+2, −*z*; (iv) −*x*+2, *y*+1/2, −*z*+1/2; (v) *x*+1, *y*, *z*.
