# Supplementary figures and images for: Crystal structure of benzimidazolium salicylate
Source: Acta Crystallogr E Crystallogr Commun. 2015 Sep 26;71(Pt 10):o794–5. doi: 10.1107/S2056989015017764 (PMC4647433; doi:10.1107/S2056989015017764)

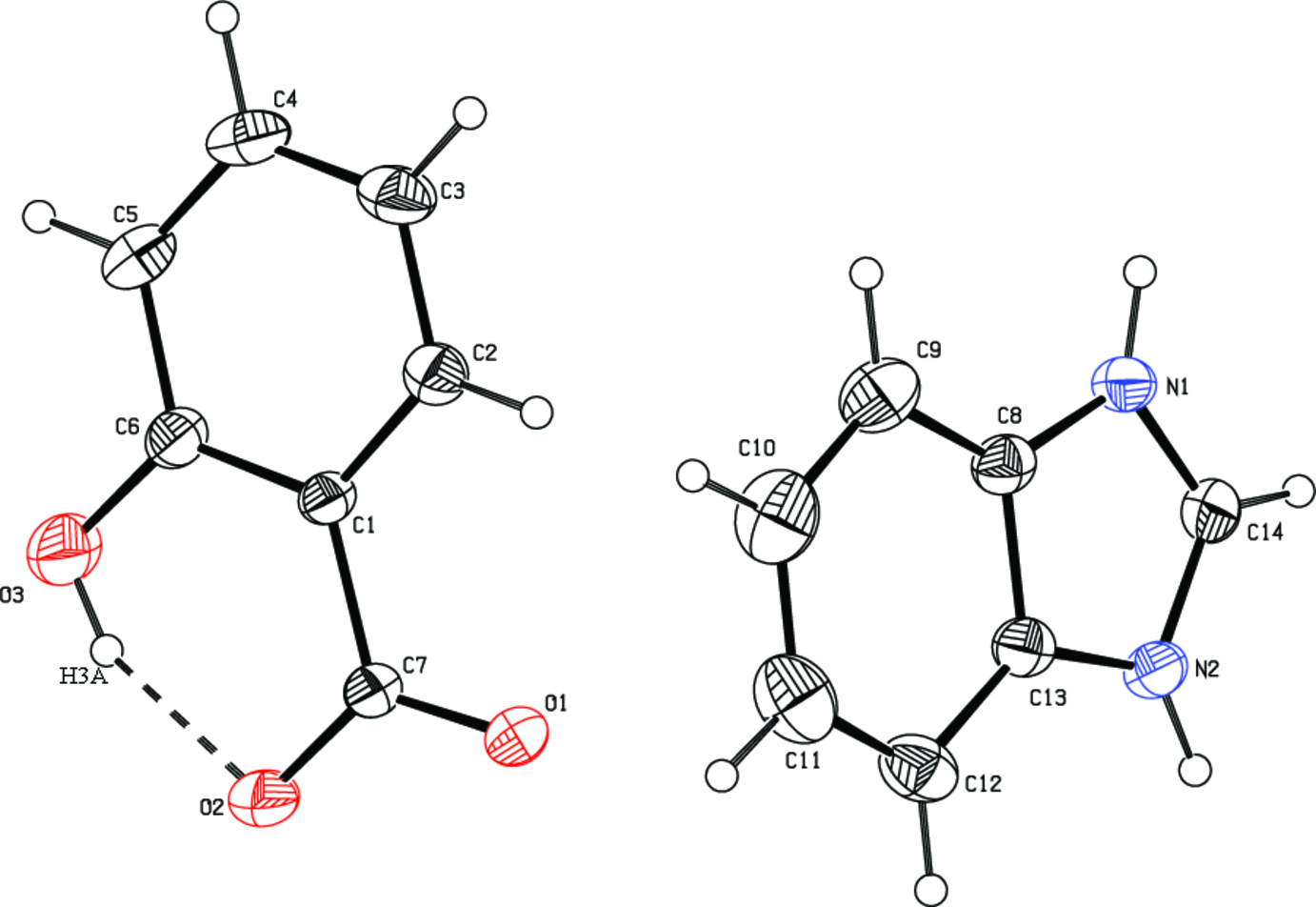

Supplement: Supplementary file 4 [file e-71-0o794-fig1.tif]

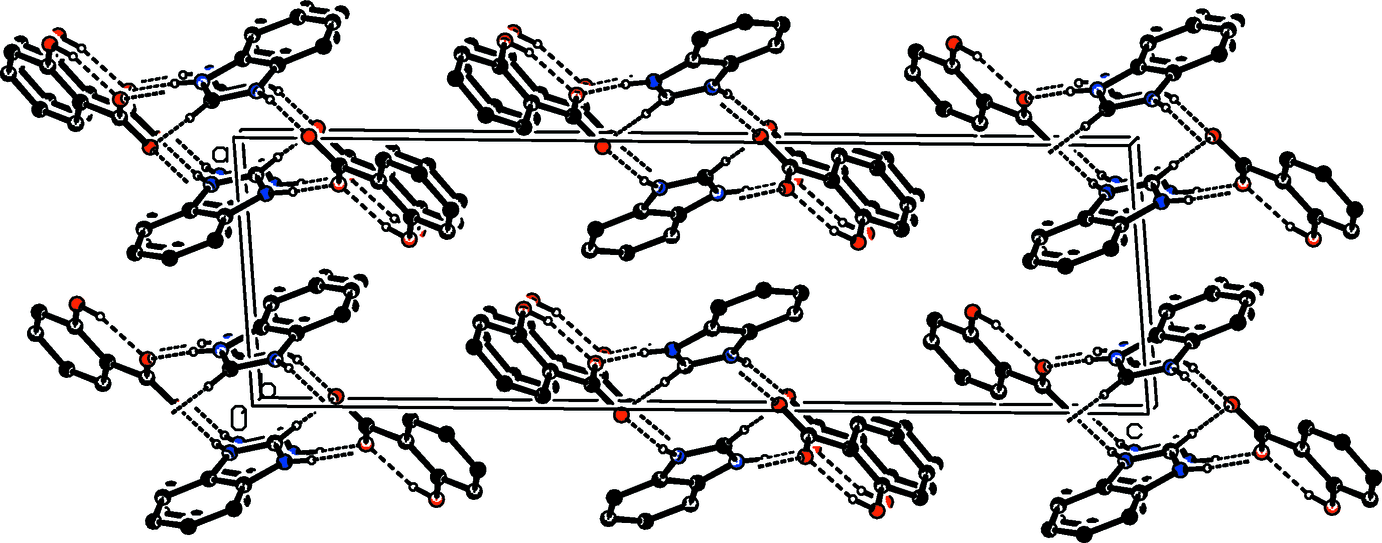

Supplement: Supplementary file 5 [file e-71-0o794-fig2.tif]
